# Massive Pulmonary Embolism With Negative D-dimer: A Case Report

**DOI:** 10.7759/cureus.76270

**Published:** 2024-12-23

**Authors:** Usamah Al-Anbagi, Abdulrahman Saad, Tarek Ibrahim, Abdulqadir J Nashwan

**Affiliations:** 1 Internal Medicine, Hamad Medical Corporation, Doha, QAT; 2 Medicine, Ministry of Public Health, Doha, QAT; 3 Pharmacy, Hamad Medical Corporation, Doha, QAT; 4 Nursing & Midwifery Research, Hamad Medical Corporation, Doha, QAT

**Keywords:** acromegaly, anticoagulation, computed tomography (ct scan), d-dimer, pulmonary embolism (pe), rivaroxaban

## Abstract

Pulmonary embolism (PE) is a critical condition that arises when clots migrate to the lungs, obstructing pulmonary circulation and posing a significant risk to the patient's health. While the D-dimer test is useful for excluding PE, it is not infallible. This report describes a case where extensive PE was present despite the patient having a normal D-dimer level, emphasizing the importance of a thorough clinical evaluation. Our case is that of a 36-year-old male patient with a known history of acromegaly. He presented to the emergency department with a cough, shortness of breath, and high-grade fever and was ultimately diagnosed with a massive bilateral PE. Despite a negative D-dimer result and a low probability based on the Wells score, the diagnosis was confirmed by a CT pulmonary angiogram. This case report raises questions about the sensitivity and safety of a negative D-dimer result in ruling out acute PE. Clinical judgment, combined with imaging, is essential for an accurate diagnosis in high-risk cases to avoid missing life-threatening conditions like PE.

## Introduction

Acute pulmonary embolism (PE) is a critical and potentially life-threatening condition resulting from the obstruction of pulmonary arteries by blood clots, typically originating from the deep veins of the legs or other parts of the venous system. It represents one of the most significant causes of cardiovascular mortality, contributing to 5-10% of hospital deaths [1]. PE is also one of the leading causes of preventable in-hospital mortality. This emphasizes the importance of timely and accurate diagnosis [2]. Venous thromboembolism (VTE), including PE and deep vein thrombosis (DVT), is a leading global cause of death, with mortality rates ranging from 9.4 to 32.3 per 100,000 people [3]. The D-dimer test, known for its high sensitivity, is commonly used to exclude PE in low and intermediate-risk patients by detecting elevated fibrin degradation products [4,5].

This case addresses the gap in PE diagnostic practices, highlighting that while the D-dimer test is functional, it may fall short in high-risk cases. It emphasizes the need for a comprehensive diagnostic approach, combining clinical assessment and imaging to diagnose and manage PE accurately. This approach helps refine diagnostic strategies and improve patient outcomes by revealing limitations in the current testing methods.

## Case presentation

A 36-year-old gentleman, diagnosed with acromegaly in 2018 due to a growth hormone-secreting pituitary adenoma, presented with acute shortness of breath, high-grade fever, chest pain, and cough of a day's duration. Despite undergoing trans-sphenoidal hypophysectomy in 2018, his symptoms of acromegaly persisted. The onset of dyspnea was sudden, accompanied by high fever and a productive cough with whitish sputum but no hemoptysis.

The patient had no history of recent travel, surgery, prolonged immobility, and no past or family history of thrombotic events. On examination, he was normotensive (127/62 mmHg), febrile (39.4°C), and tachypneic (21 breaths per minute), and had tachycardia (123 beats per minute) which settled to 80 beats per minute after his temperature returned to normal. His oxygen saturation was 87% on room air, requiring supplemental oxygen at five liters per minute.

**Figure 1:**
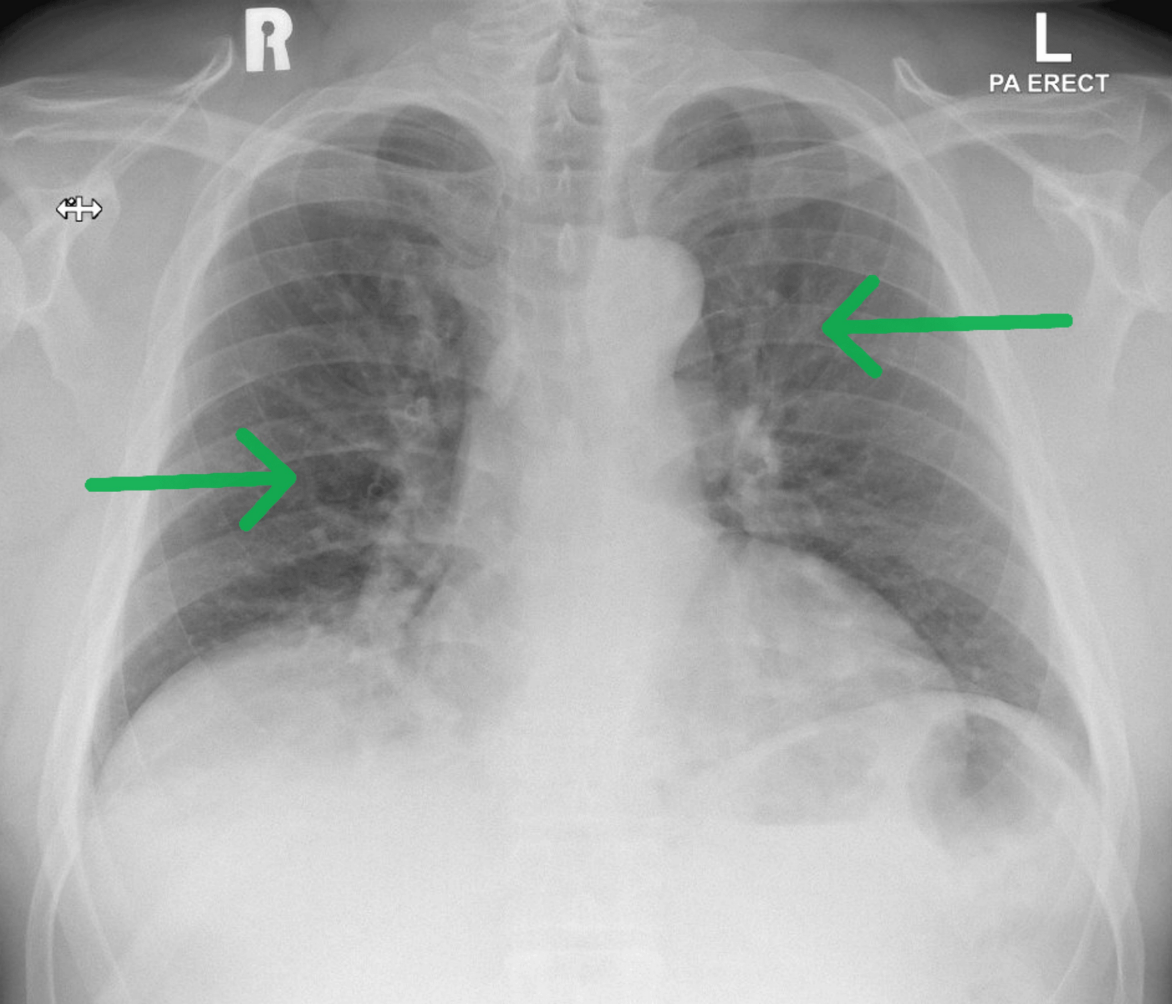
Chest X-ray showed increased bronchovascular markings

Physical examination revealed typical features of acromegaly, including coarse facial features, frontal bossing, a large and wide nose, prognathism, spade-shaped hands, large feet, a harsh voice, and skin folding. Systemic examination, including visual field and acuity tests, were unremarkable. Chest X-ray showed increased bronchovascular markings but was otherwise normal (Figure 1).

**Figure 2:**
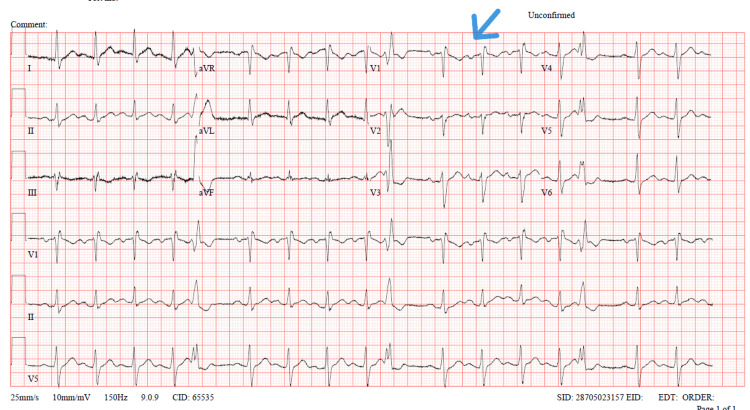
Electrocardiogram showing incomplete right bundle branch block. The blue arrow shows rSR and QRS complex duration around 120 ms in V1, with normal R peak in V6, indicating incomplete right bundle branch block (RBBB).

Electrocardiography indicated sinus tachycardia and right bundle branch block. Echocardiography revealed moderately increased right ventricular systolic pressure, suggesting right ventricular strain. The ECG showed an incomplete right bundle branch block (Figure 2).

Initial blood tests revealed mild leukocytosis, slightly elevated C-reactive protein (CRP), and a negative D-dimer (Table 1).

**Table 1:** Laboratory results FEU, Fibrinogen equivalent units; ANA, Antinuclear antibody; Anti dsDNA, Anti double-stranded DNA; Anti RO52, Anti-tripartite motif-containing 21; Anti-SSA, Anti-Sjögren's-syndrome-related antigen A; Anti-Sm, Anti-Smith; Anti-RNP, Anti-ribonucleoprotein; Anti PCNA, Anti-proliferating cell nuclear antigen; Anti-MA-M2, Anti-mitochondrial M2; Anti PM-Scl, Anti-polymyositis/scleroderma; AST, Aspartate aminotransferase; ALT, Alanine aminotransferase; TSH, Thyroid stimulating hormone; FT4, Free thyroxine; INR, International normalized ratio.

Parameters	Patient values	Reference values
D-dimer (mg/L FEU)	0.39	0-0.49
Anti-cardiolipin IgG antibody (U/ml)	0.9	<15
Anti-cardiolipin IgM antibody (U/ml)	2.2	<10
ANA profile (includes Anti dsDNA, Anti RO52, Anti-SSA, Anti nucleosomes, Anti-Sm, Anti RNP, Anti histones, Anti PCNA, Anti SS-B, Anti ribosomal-P-protein, Anti-JO-1, Anti AMA-M2, Anti centromere B, Anti PM-Scl antibodies)	All negative	Negative
Factor II	Normal	Normal
Factor V	Normal	Normal
Lupus screen (seconds)	36.4	30.4-45.3
Lupus anti-coagulant	Not detected	Not detected
Protein C activity	110.6%	70-140%
Protein S activity	99.5%	72-126%
Anti-thrombin activity	130%	79.4-130%
Fibrinogen (gm/L)	4.9	2-4.1
INR	1.2	<1.1
Prothrombin time (seconds)	13.3	9.5-12.5
Total leukocytes (x10^3^/µL)	11.2	6.2
Serum hemoglobin (gm/dl)	13.3	13-17
Hematocrit	37.8%	40-50%
Serum potassium K (mmol/L)	3.7	3.5-5.3
Serum sodium (mmol/L)	138	133-146
Serum magnesium (mmol/L)	0.72	0.7-1
Serum urea (mmol/L)	3.7	2.5-7.8
Serum creatinine (umol/L)	67	62-106
Serum glucose (mmol/L)	8	<11.1
Serum albumin (gm/L)	35	35-50
Serum total protein (gm/L)	72	60-80
AST (IU/L)	22	0-41
ALT (IU/L)	34	0-41
Alkaline phosphatase (U/L)	115	40–129
TSH (mIU/L)	0.69	0.3-4.2
FT4 (pmol/L)	13.6	11-23.3
Serum total bilirubin (mg/dl)	11	0-21
Serum morning cortisol	240	138-689

**Figure 3:**
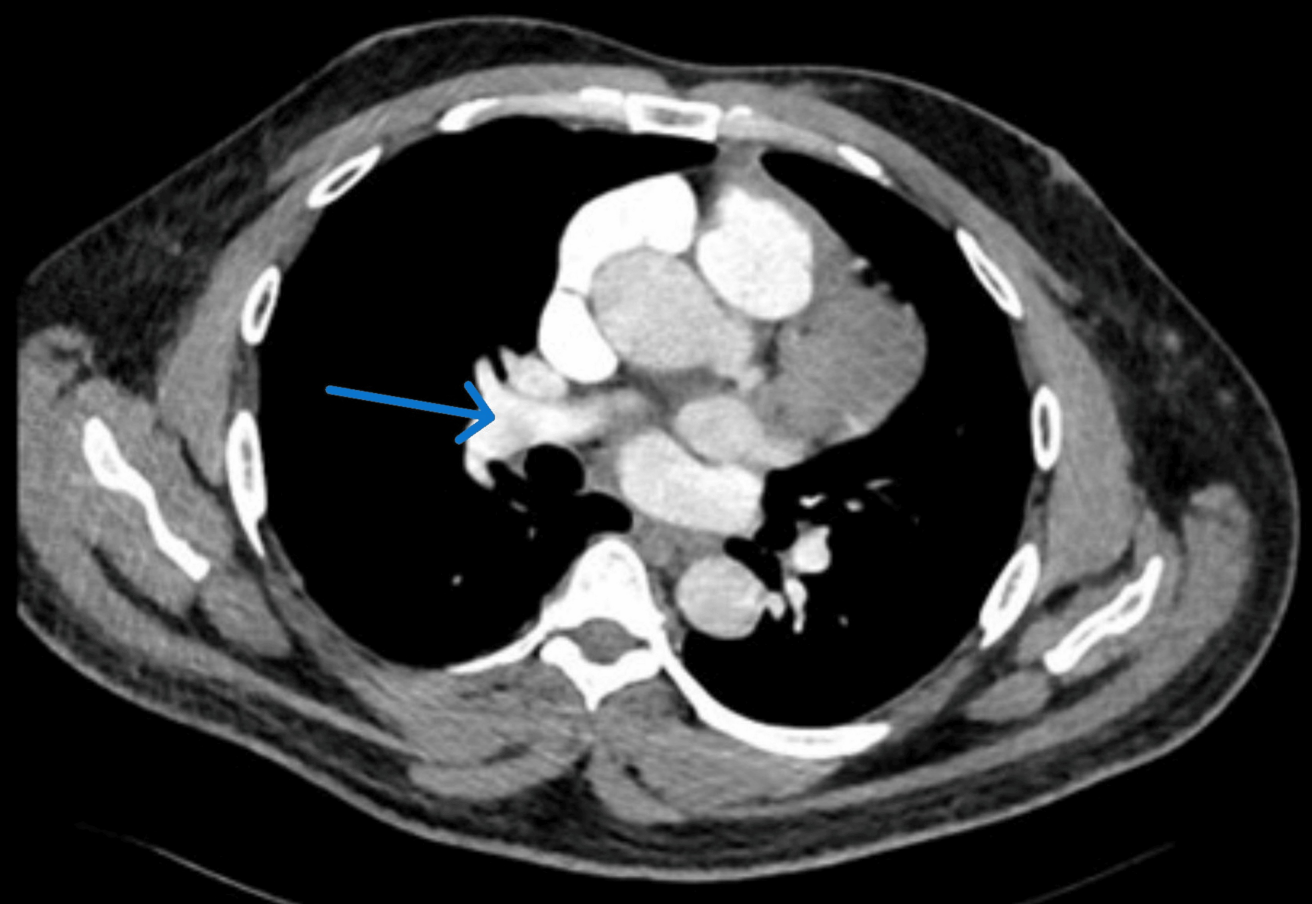
Computed tomography pulmonary angiogram showing bilateral partial filling defect (blue arrow) indicating pulmonary embolism (right side)

**Figure 4:**
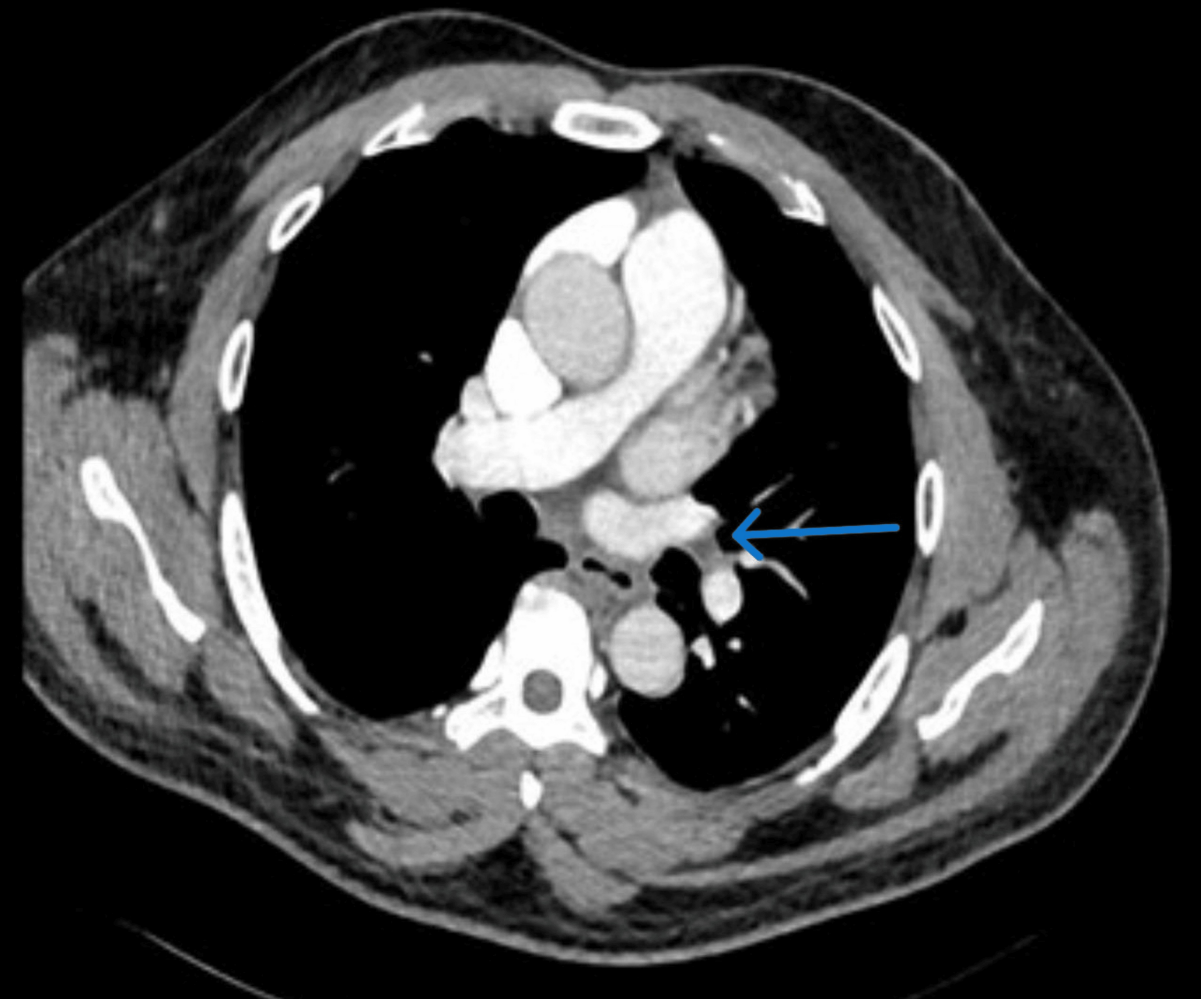
Computed tomography pulmonary angiogram showing bilateral partial filling defect (blue arrow) indicating pulmonary embolism (left side)

Despite the negative D-dimer result, high clinical suspicion led to an urgent computed tomography pulmonary angiogram (CTPA), which confirmed a bilateral massive PE (Figures 3, 4).

The patient was promptly started on therapeutic subcutaneous low-molecular-weight heparin and transferred to the high-dependency unit. After consulting endocrinology and pulmonology, the patient was transitioned to rivaroxaban [a direct oral anticoagulant (DOAC)] and continued treatment for his diabetes and hypertension. After treatment, the patient improved and was discharged in a stable condition on rivaroxaban, with plans for outpatient follow-up at the clinic.

## Discussion

PE is a life-threatening condition, and although rare, its occurrence in patients with acromegaly is a critical consideration [5]. The European Society of Cardiology (ESC) classification of PE categorizes it into low, intermediate (sub-massive), and high (massive) risk based on hemodynamic stability, signs of right ventricular dysfunction, myocardial injury, and risk of mortality, guiding treatment decisions from anticoagulation to thrombolysis or surgery [5].

Acromegaly is characterized by excessive growth hormone secretion and results in various systemic complications, such as an increased risk of thromboembolic events due to alterations in coagulation factors. This prothrombotic state is driven by elevated levels of fibrinogen, factor VII, and plasminogen activator inhibitor-1 (PAI-1), all contributing to a higher risk of arterial and venous thromboembolism [6].

In 1912, Coffey and Cummins were among the first to propose a link between acromegaly and an elevated risk of thrombosis. This was based on a case where a patient succumbed to PE and thrombosis [7]. Subsequent case reports have also documented instances of VTE in patients with acromegaly despite the absence of other identifiable risk factors. Those reports included cases where acromegaly was flagged at the time of diagnosis of the thrombotic event or a pre-existing condition managed inadequately [8-10].

This case report describes a 36-year-old male patient with acromegaly who presented with acute shortness of breath, chest pain, and cough and was ultimately diagnosed with massive bilateral PE despite a negative D-dimer test. The complexity of this case highlights several important considerations in managing patients with acromegaly and suspected PE.

Studies indicate that patients with acromegaly have a two- to threefold increased risk of VTE compared to the general population. Colak et al. (2012) found that patients with acromegaly have a higher risk of arterial and venous thromboembolic events. Their research suggested that the prothrombotic state associated with acromegaly can persist even after the disease is biochemically controlled, making VTE a potential long-term complication. Despite treatments like hypophysectomy, thrombotic risks can remain, as demonstrated by a case where acromegaly persisted after surgery [11].

D-dimer is a marker of blood clot breakdown, often used to diagnose thrombosis, including PE. While D-dimer levels are typically elevated in acute PE, there is a debate over the optimal threshold and its usefulness due to other conditions that can also raise D-dimer levels. Some studies suggest that D-dimer could reduce the need for CTPA scans in PE screening [12]. An elevated D-dimer level alone cannot diagnose PE, but a normal D-dimer can help rule out PE in patients with low or intermediate risk. A normal D-dimer (<500 ng/mL) effectively excludes PE in low-risk patients, while an elevated D-dimer requires further imaging. In intermediate-risk patients, a normal D-dimer also typically excludes PE, though some experts suggest imaging for those with higher intermediate risk due to lower D-dimer sensitivity. For high-risk patients, a normal D-dimer is less useful, as it cannot sufficiently rule out PE, and these patients should undergo imaging, preferably with CTPA, even if the D-dimer test is negative [13,14].

A study of 2017 patients found that 7.4% had PE on initial testing. Among 1325 patients with low or moderate clinical probability of PE and negative D-dimer tests, none developed VTE during follow-up. In a study of 1863 patients without an initial PE diagnoses and not on anticoagulants, only one developed VTE (0.05%). Using the current diagnostic strategy, chest imaging was required in 34.3% of patients, compared to 51.9% if PE was ruled out with lower D-dimer thresholds. The results support ruling out PE in patients with low clinical pretest probability (C-PTP) and D-dimer below 1000 ng/mL and those with moderate C-PTP and D-dimer below 500 ng/mL [15].

CTPA and magnetic resonance pulmonary angiography (MRPA) are the main diagnostic tools for PE, and they identify a filling defect in the pulmonary arteries after contrast enhancement. Ventilation-perfusion (V/Q) scanning can also diagnose PE when a perfusion defect is present with normal ventilation, and a normal or low-probability scan excludes PE in low-risk patients. High-probability scans confirm PE in high-risk patients. Catheter-based pulmonary angiography is diagnostic when a vessel filling defect is seen. Echocardiography can help in unstable patients but is rarely diagnostic. Lower-extremity ultrasound detects DVT, which may guide treatment but doesn't diagnose PE [16]. 

The management strategy involved the immediate initiation of therapeutic anticoagulation with low molecular weight heparin, followed by transition to rivaroxaban. This approach aligns with the current guidelines recommending prompt anticoagulation in cases of confirmed massive PE to prevent further thrombotic complications and improve outcomes [17].

## Conclusions

This case highlights the importance of combining clinical probability assessments with diagnostic tests, such as D-dimer and CTPA, for accurate diagnosis of PE. While D-dimer testing can effectively rule out PE in low and intermediate-risk patients, imaging is crucial for confirmation, especially in high-risk cases. Despite a negative D-dimer result, this case underscores the limitations of D-dimer testing, as the patient was ultimately diagnosed with massive PE. It also emphasizes the need for strong clinical judgment to avoid overlooking a life-threatening condition.
